# Household Knowledge

**Published:** 1873-08

**Authors:** 


### HOUSEHOLD KNOWLEDGE.

A SINGLE drop of the sesquipede chloride of iron, put on a corn between the toes, once a day, with a camel's hair brush, will effect a marvellous cure.

Ten grains of oxalic acid in half a pint of water, will remove all ink and fruit stains. Wet the article in hot water, and apply it to the top of the bottle, so that the liquid will reach it, then rinse it well.

Dry paint is removed by dipping a swab with a handle in a strong solution of oxalic acid. It softens it at once.

Common ley of wood ashes will soften hard putty in a few minutes.

Keep some strong spirits of hartshorn in a ground glass stoppered bottle ; a teaspoonful in a tablespoonful of water will clean combs and brushes, and restore colors injured by acids. A weaker solution, applied to ill smelling feet and arm-pits, removes the odor, and removes grease spots from carpets and clothing. A weak solution in water makes a good wash for the hair, and stimulates its growth when impaired by fever, and cleanses the scalp effectually. A weak solution scattered over the leaves of plants, from a soft, fine, limber brush, gives new life to plants. Even if a little is sprinkled over the earth at their roots their growth is invigorated.

Borax, half a teaspoonful, in half a teacup of water, makes the mildest and most efficient hair and scalp cleaner in the world. Rub it into the scalp with the balls of the fingers, head held over a basin, eyes shut, until the entire scalp is in a foam, then rinse with warm water.

The fumes of a brimstone match will remove berry stains from a book, or paper, or engraving.

If a door does not shut without a "slam," put a drop of sweet oil on the catch, or on the hinge, if it creaks. Soap will do, but not so well.

If there is rust on your flat iron, or other roughness, put some fine salt on a board and rub it rapidly while warm, until it moves smoothly.

If rats are about, scatter powdered glass about their holes, or powdered copperas, or fill up the crevices with hard soap, or smear their holes with soft tar, or dip the rat in a cup of tar and let it go, and it will tar plaster every hole in the house.

If you wish to make a nail drive easily and last long without rusting, dip it in melted grease first. This is excellent for fencing and other exposed work.

If a glass stopper won't move, hold the neck of the bottle to a flame, or warm it by taking two turns of a string and see-saw it; the heat engendered expands the neck of the bottle before a corresponding expansion reaches the stopper.

If you want a new shoe to fit as easily as an old one, put on two pair of stockings before your measure is taken.

